# Cellular and Molecular Mechanisms of In Vivo and In Vitro SARS-CoV-2 Infection: A Lesson from Human Sperm

**DOI:** 10.3390/cells11172631

**Published:** 2022-08-24

**Authors:** Alice Luddi, Francesca Paola Luongo, Filippo Dragoni, Lia Fiaschi, Ilaria Vicenti, Pietro Lupetti, Mariangela Gentile, Eugenio Paccagnini, Alesandro Haxhiu, Rosetta Ponchia, Laura Governini, Maurizio Zazzi, Paola Piomboni

**Affiliations:** 1 Department of Molecular and Developmental Medicine, University of Siena, 53100 Siena, Italy; 2 Department of Medical Biotechnologies, University of Siena, 53100 Siena, Italy; 3 Department of Life Sciences, University of Siena, 53100 Siena, Italy

**Keywords:** SARS-CoV-2, COVID-19, human sperm, human testis, male infertility

## Abstract

Despite the major target of Severe Acute Respiratory Syndrome Coronavirus 2 (SARS-CoV-2), the causative agent of COVID-19, being the respiratory system, clinical evidence suggests that the male reproductive system may represent another viral target organ. Revealing the effect of SARS-CoV-2 infection on testis and sperm is a priority for reproductive biology, as well as for reproductive medicine. Here, we confirmed that the SARS-CoV-2 receptor angiotensin-converting enzyme 2 (ACE2) is highly expressed on human testis and ejaculated sperm; moreover, we provide evidence for the expression of the co-receptors transmembrane protease/serine (TMPRSS2), Basigin (BSG), and Catepsin L (CTSL). Human sperm were readily infected, both in vivo and in vitro, by SARS-CoV-2, as demonstrated by confocal and electron microscopy. The demonstration that the seminiferous epithelium and sperm support SARS-CoV-2 viral replication suggests the possibility that the spermatogenetic process may be detrimentally affected by the virus, and at the same time, supports the need to implement safety measures and guidelines to ensure specific care in reproductive medicine.

## 1. Introduction

The SARS-CoV-2 pandemic has already affected millions of people worldwide, with an impressive impact on medical care in general and also on the delivery of infertility treatments. A recent study shed light on the impact of SARS-CoV-2 on female fertility, in particular the microfollicular environment, pointing out the vulnerability of the reproductive system to infection [1]. Further studies on male reproduction are needed. A growing body of evidence supports the idea that males are more vulnerable to SARS-CoV-2 disease than females, with a higher mortality rate [2,3]. However, the reasons for gender-related differences in the outcome of COVID-19 remain to be defined. Interestingly, orchitis has been reported to be a complication of SARS [4], and free testosterone levels are inversely correlated with prognostic factors of severe COVID-19 [5]. This advises monitoring spermatogenesis and, more generally, male fertility in acute and convalescent COVID-19 patients.

To enter target cells, SARS-CoV-2 uses the S protein, which binds to the angiotensin-converting enzyme 2 (ACE2). Specific host proteases, including cell surface transmembrane protease/serine (TMPRSS2) proteases, cathepsins, basigin, elastase, and trypsin, are also involved in the cleavage of the S protein, which activates ACE2-mediated virus entry by facilitating fusion between the virus envelope and the host cell plasma membrane [6,7].

Numerous data support the idea that SARS-CoV-2 can bind to and infect male germ cells and testicular somatic cells, including Sertoli and Leydig cells. ACE2 was highly expressed in spermatogonia, Leydig cells, and Sertoli cells [8,9], while TMPRSS2 is not often co-expressed in testicular cells and sperm [10]. However, TMPRSS2 expression is promoted by the androgen response element, and circulating androgen levels in men are positively correlated with TMPRSS2 expression [11]. The expression of cathepsins L (CTSL) and basigin (BSG) are also evident in testes, epididymis, and seminal vesicles [12].

Despite this wide expression of SARS-CoV-2 receptors, the presence of the virus inside the sperm cells of infected men is still debated. While some authors did not detect the virus in human semen, either in the acute phase or the convalescent phase [13,14,15,16], other studies detected the virus in semen samples of patients with a high viral load during the acute or convalescent phase of infection [17,18].

In this study, we demonstrated, by different, comprehensive approaches, the expression of key molecules (ACE2, TRPMSS2, BSG, and CTSL) responsible for viral entry into host cells, both in human testis and in ejaculated sperm. Using immunoelectron microscopy, qRT-PCR, and immunofluorescence, we provide strong evidence for the ability of SARS-CoV-2 to infect human sperm in vitro.

## 2. Materials and Methods

### 2.1. Human Biological Sample Collection

All the participants provided written informed consent for the use of their samples and data. This study was approved by the University of Siena’s ethics committee (CEAVSE, protocol number 18370, 2/10/2020). For the in vitro studies, we enrolled a total of 20 Caucasian males aged 30 ± 4.5 years undergoing semen evaluation at the Unit of Medically Assisted Reproduction at Siena University Hospital. A comprehensive clinical history was obtained for all participants, and subjects with possible preexisting causes of male infertility, such as varicocele, cryptorchidism, or endocrine disorders, were excluded. In addition, testis samples were obtained from 10 patients aged 31 ± 2.5 years undergoing diagnostic testicular biopsies for obstructive azoospermia. None of these patients received chemotherapy, radiotherapy, or hormonal treatment. Upon testicular biopsies, around 5 mm^3^ of tissue was cryopreserved in RNA later (Qiagen, Hilden, Germany) and stored at −80 °C. 

This study also included the ultrastructural analysis of sperm from a 40-year-old man diagnosed with mild COVID-19 infection based on positivity on SARS-CoV-2 RT-PCR from nasopharyngeal swabs obtained two days before sperm collection. The patient reported several symptoms, including fever, cough, nasal congestion, headache, and muscle pain.

### 2.2. Sample Collection and Analysis

Semen samples were obtained by masturbation after 3–5 days of sexual abstinence. Standard semen analysis was performed according to WHO protocol [19], and all subjects enrolled for the in vitro studies were characterized for main sperm parameters, namely concentration, morphology, progressiveness, and total motility [20]. Sperm samples were then treated for TEM analysis or for immunofluorescence or stored at −80 C° for gene expression analysis.

### 2.3. In Vitro Sperm Infection by SARS-CoV-2

Sperm cell infection was performed in duplicate, collecting a minimum of 5 × 10^8^ sperm cells from two patients. The SARS-CoV-2 strain EPI_ISL_2472896, belonging to the lineage B.1, was kindly provided by the Department of Biomedical and Clinical Sciences “Luigi Sacco” at the University of Milan (PMID 34391446). The viral stock used to infect sperm cells was kept at −80 °C and titrated by plaque assay in VERO E6 cells (ATCC^®^ CRL-1586), as previously described (PMID 34273661). All procedures related to virus cultures were carried out in a biosafety level 3 facility, according to the WHO guidelines. Once counted, sperm cells were aliquoted in 3 tubes and subjected to different infection procedures before swim-up:

Infection with SARS-CoV-2 viral stock at the multiplicity of infection (MOI) 0.5 with virus adsorption for 2 h at room temperature (ADS);

Infection under the same condition but without virus adsorption (No_ADS);

Incubation for 2 h at room temperature without virus (cell control).

Following infection, the sperm cell preparations were centrifuged, and the bottom 100 µL underwent density gradient centrifugation by using an 80–40% Pure Sperm gradient (Nidacon, Mölndal, Sweden) to purify sperm from debris and any other somatic cells. After washing with PBS 1x 200 µL of cell pellet was counted to normalize the gene expression results as SARS-CoV-2 RNA copies per million sperm cells and/or per millilitre. In addition, a virus control simulating the initial viral inoculum was subjected to the same procedure in the absence of sperm cells to estimate the amount of downstream residual virus.

### 2.4. RNA Extraction and One-Step Digital Droplet PCR (ddPCR)

Total RNA was isolated by RNeasy Protect Mini kit (Qiagen, Hilden, Germany) from both sperm cells and frozen testis biopsies following tissue that was homogenized, as previously mentioned [21]. At the end of the procedure, RNA was eluted in a final volume of 30 µL of RNase-free water. 

The purity and the concentration of RNA were evaluated by readings on the Nano Drop^®^ ND-100 UV-vis Spectrophotometer (Nano Drop Technologies, Waltham, MA, USA). RNA samples were subjected to DNAse treatment (Sigma-Aldrich, Burlington, MA, USA), and gene expression analysis was performed using specific EvaGreen assays (Table S1) by QX200 droplet digital (dd)-PCR System according to the manufacturer’s instructions (Bio-Rad, Hercules, CA, USA). The cycling conditions are summarized in Supplementary Table S2. Samples were amplified with the following cycling conditions: 42 °C for 60 min and 95 °C for 10 min, then 39 cycles at 95 °C for 30 sec and 59 °C for 1 min, and finally 98 °C for 10 min. After PCR, the 96-well PCR plate was loaded into the QX200 Droplet Reader (Bio-Rad, CA, USA) to identify the fluorescence intensity of each droplet for the EvaGreen fluorophore. Data were analyzed using the QuantaSoftTM Analysis Pro software, version 1.0 (Bio-Rad, CA, USA). A threshold line was employed to discriminate positive and negative droplets. The Poisson statistics were applied to calculate the absolute concentration of each target gene in copies/µL. The reference genes GAPDH and PPBI were used to normalize RNA amount; the relative gene expression was expressed as normalized sample amount (NSA). 

### 2.5. Detection of SARS-CoV-2 RNA

Viral RNA extraction was performed from 200 µL of infected cell pellets (ADS and No_ ADS), cell control, and virus control using the ZR Viral RNA kit (Zymo Research, #R1035) according to the manufacturer’s instructions. Viral RNA quantification was performed using Bio-Rad’s QX200 ddPCR System (Bio-Rad, Hercules, CA, USA, #1864001) according to the manufacturer’s instructions. Primers and probes were designed on the NC_045512.2 reference strain targeting the SARS-CoV-2 polybasic cleavage site in the spike region as previously described [1]. ddPCR was performed in a total volume of 20μL, containing 4 µL of RNA extract diluted 50,000-fold, 5X One-Step RT-ddPCR. Advanced kit for probes (Bio-Rad, CA, USA, #1864021), 900 nM forward and reverse primer, 250 nM FAM-labelled probes (Table S1), and 14 mM dithiothreitol solution (Bio-Rad, CA, USA), and RNase-free sterile water were used to reach the final volume. The mixture was added to the DG8 cartridge (Bio-Rad, CA, USA, #1864008), followed by the loading of 70 µL of droplet-generation oil for probes (Bio-Rad, CA, USA, #1863005) using an automated droplet generator (Bio-Rad, CA, USA, #1864002). Then, 40 µL of generated droplets was transferred into a 96-well PCR plate and heat-sealed with a pierceable PCR Plate Heat Seal foil (Bio-Rad, CA, USA, #1814040) using a PX1 PCR Plate Sealer (Bio-Rad, CA, USA, #1814000) for 3 s at 175 °C twice and then placed in the thermal cycler (T100 Thermal Cycler, Bio-Rad, CA, USA, #FB2580). Samples were amplified with the following cycling conditions: 45 °C for 60 min and 95 °C for 10 min, then 45 cycles at 95 °C for 30 sec and 60 °C for 1 min, and finally 98 °C for 10 min. After PCR, the 96-well PCR plate was loaded into the QX200 Droplet Reader (Bio-Rad, CA, USA, #1864003) to identify the fluorescence intensity of each droplet for FAM-TaqMan probe. Data were analysed using the QuantaSoft software, version 1.7.4.0197 (Bio-Rad, CA, USA). The threshold line to discriminate positive and negative droplets was set based on the signal emitted by the control cell ddPCR sample.

### 2.6. Immunofluorescence

For immunohistochemistry, formalin-fixed, paraffin-embedded testicular biopsies were sectioned at 4 µm thickness; sections were then fixed and deparaffinized with xylene and dehydrated with ethanol as previously described with some modification [22]. Antigen retrieval was obtained by incubation in10 mM citrate buffer with pH 6.0 at a sub-boiling temperature for 20 min. Slides were left to cool for 10 min. 

Immunofluorescence on in vitro infected spermatozoa was performed after fixation with 4% PFA for 1 h, followed by three washes in PBS 1x. Once resuspended in a suitable volume, the sperm were smeared onto glass slides and left to dry [23].

For both immunohistochemistry and immunofluorescence, the slides were incubated in a blocking solution containing 5% goat normal serum in 1% PBS/BSA (bovine serum albumin) and then incubated overnight at 4 °C with ACE2, TMPRSS2, BSG, CTSL, and INSL3 primary antibodies (Table S2), carried out according to the manufacturer’s instructions. The specificity of immunostaining was confirmed by using pre-immune sera instead of the primary antibody, followed by incubation with the secondary antibody. The slides were washed in PBS, and the bound antibodies were revealed by incubation with FITC-labelled anti-rabbit and TRITC-labelled anti-mouse secondary antibodies (Table S2). After washing in PBS, the slides were mounted in ProLong antifade with 4′,6-diamidino-2-phenylindole (DAPI) (LifeTechnologies, Carlsbad, CA, USA) to counterstain the nuclei, and then they were observed with a Leica DMB 6000 immunofluorescent microscope (Leica Mycrosistem, Wetzlar, Germany). Images were captured with a CFTR6500 digital camera (Leica Mycrosistem, Wetzlar, Germany).

### 2.7. Transmission Electron Microscopy (TEM)

SARS-CoV-2 infected spermatozoa were fixed in 2.5% glutaraldehyde diluted in 0.1 M, pH 7.2 cacodylate buffer (CB) for 2 h at 4 °C, washed in CB overnight, and then postfixed in 1% osmium tetroxide for 1 h at 4 °C. Samples were dehydrated with a graded series of ethanol and embedded in Epon. Sections (70 nm thin) were obtained with a Reichert Ultracut E ultramicrotome and collected on 150 mesh copper grids. Sections were stained with uranyl acetate and lead citrate and observed with an FEI Tecnai G2 Spirit transmission electron microscope operating at an electron accelerating voltage of 120 kV and equipped with a TVIPS TemCam-F216 CMOS camera.

### 2.8. Statistic Analysis

Statistical analysis was performed using GraphPad Prism 5.0 (GraphPad Software, San Diego, CA, USA). The nonparametric Kruskal–Wallis test and the Mann–Whitney test were used to evaluate the statistical difference among groups of data. Statistical significance was set at *p* < 0.05.

## 3. Results

**Figure 1:**
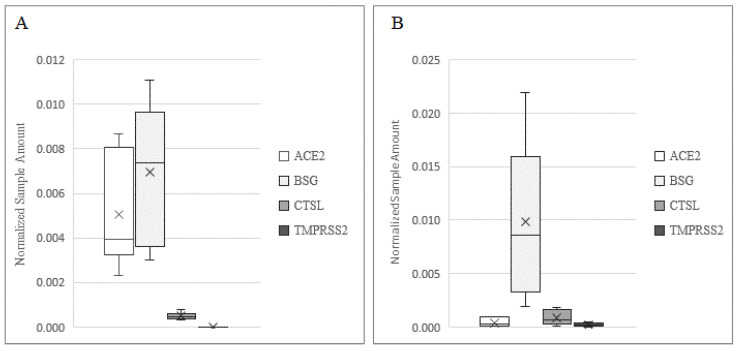
Expression of *ACE2*, *BSG*, *CTSL,* and *TMPRSS2* in human testis (**A**) and in ejaculated sperm. (**B**) Data are plotted as box–whisker plots, where boxes show the interquartile range with median and mean values, and whiskers represent min and max confidence intervals. Data were derived from 20 testicular biopsies and 10 sperm samples.

In order to demonstrate the mechanism of infection of SARS-CoV-2 in human testis and sperm, we investigated the presence of receptors and co-receptors using different methods (Figure 1). BSG was detected at the highest levels in both the testis and sperm, while ACE2 was expressed at a higher level in the testis as compared to sperm (*p* < 0.001). The expression profile of CTSL in the two samples was comparable, while TMPRSS2 was expressed at a low level in sperm and was barely detectable in testis biopsies (*p* < 0.001).

**Figure 2:**
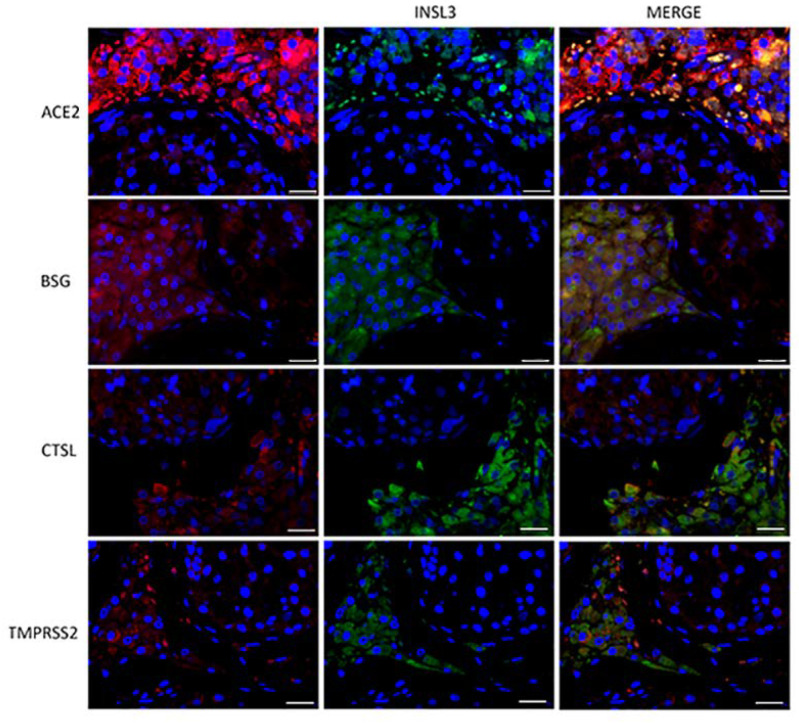
Double-staining immunofluorence of the ACE2 SARS-CoV-2 receptor and the other relevant host factors, BSG, CTLS, and TMPRSS2 (in red), and their colocalization with INSL3, a specific marker of Leydig cells (in green). Scale bar = 25 μm.

To evaluate the subcellular localization of the corresponding proteins, immunofluorescence analysis of both testis sections and sperm was performed. ACE2, BSG, CTSL, and TMPRSS2 were strongly co-localized with INSL3, a marker of Leydig cells (Figure 2). BSG and CTSL also showed less-intense staining in the seminiferous epithelium, likely in the Sertoli cells. Compared to the other co-receptors, TMPRSS2 confirmed a lower intensity and a localization restricted to the Leydig cells in the interstitium (Figure 2).

**Figure 3:**
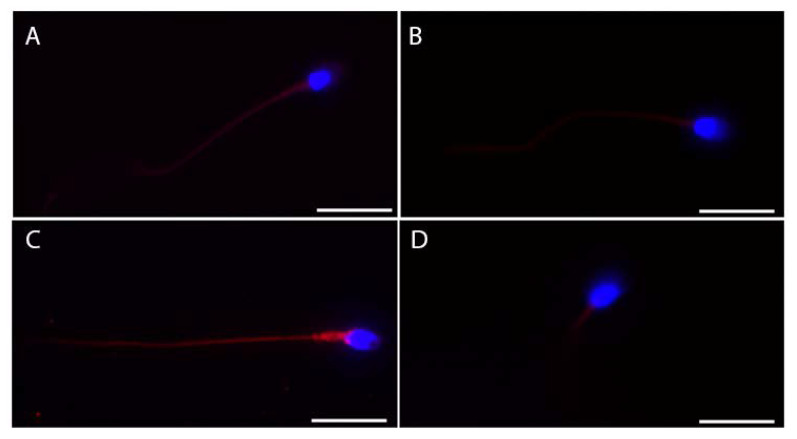
Representative micrographs (from three independent experiments) of immunofluorescence staining and confocal microscopy for the SARS-CoV-2 receptor ACE2 (**A**) and the other relevant host factors, BSG (**B**), CTSL (**C**), and TMPRSS2 (**D**), in ejaculated human sperm. ACE2, BSG, CTLS, and TMPRSS2 are in red; nuclei are in blue. Scale bar = 15 µm.

We also tested the subcellular localization of these proteins in ejaculated sperm (Figure 3). ACE2 showed more intense staining in the whole tail (Figure 3A), while faint staining at the midpiece level of the sperm tail was detectable for both BSG and TMPRSS2 (Figure 3B,D). Finally, CTSL showed the most intense and bright staining of the whole tail of the ejaculated sperm (Figure 3C).

### Quantification of SARS-CoV-2 RNA Associated with Sperm Cells after In Vitro Infection

To compare the amount of SARS-CoV-2 RNA in infected sperm cells with or without viral absorption (ADS and No_ADS), values were expressed as viral RNA copies per million sperm cells. To evaluate the difference between the virus control and the ADS or No_ADS sperm cells, values were expressed as RNA copies per millilitre because virus control was run in the absence of sperm cells and thus could not be normalized per million sperm cells.

**Figure 4:**
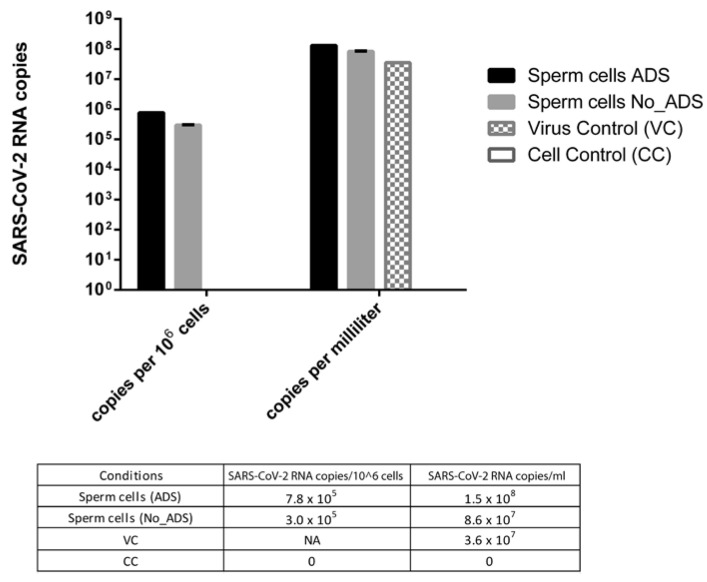
SARS-CoV-2 RNA amount in infected sperm cells with (ADS) or without (NO_ADS) viral absorption, expressed as viral RNA copies per million sperm cells and as viral RNA copies per millilitre. The virus control (VC) subjected to swim-up in the absence of sperm cells to estimate the residual viral inoculum was expressed only as RNA copies per millilitre. CC: uninfected sperm cells, NA: not applicable.

As shown in Figure 4, the viral RNA titer was 2.6-fold higher in the ADS with respect to the No_ADS sperm cells, suggesting that the viral-adsorption step increased the physical association between the virus and sperm cells. In addition, the viral RNA titer was higher in the sperm cells (4.0- and 2.4-fold for ADS and No_ADS, respectively) with respect to the virus control, confirming that the ddPCR signal from sperm cells derived from the specifically bound virus is distinct from the residual virus inoculum.

**Figure 5:**
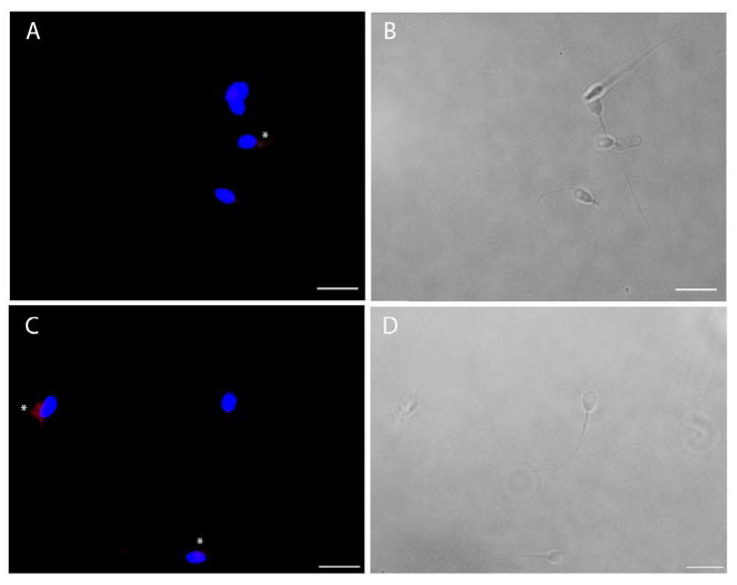
Intracellular immunofluorescence localization of SARS-CoV-2 nucleocapsid (**A**) and spike (**C**) proteins and bright filed images (respectively **B** and **D**) of ejaculated human sperm in vitro infected by SARS-CoV-2. The fluorescent staining is localized in the cytoplasmic residues at the midpiece level (asterisks). Nuclei were counterstained with 4,6-diamino-2-phenylindole (DAPI) (blue). Scale bar = 5 µm.

The viral infection of human sperm was furthermore confirmed by immunofluorescence for spike and nucleocapsid viral proteins (Figure 5). For both viral antigens, about 30% of cells were positive (Figure 5), particularly in cases of cytoplasmic residues, both at the head and midpiece tail level. 

**Figure 6:**
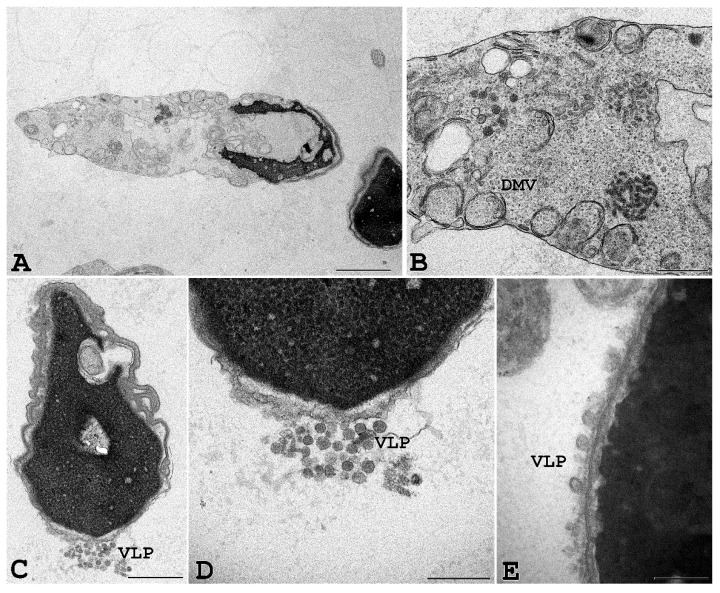
Ejaculated human sperm infected in vitro with SARS-CoV-2. Viral-like particles (VLP) and double-membrane vesicles (DMV) are seen in the sperm, particularly in cytoplasmic residues embedding the sperm head and/or tail. Scale bar: (**A**) and (**D**) = 1 µm; (**B**) = 2 µm, (**C**) = 0.5 µm; (**E**) = 0.2 µm.

To further confirm that SARS-CoV-2 can penetrate human spermatozoa in vitro, we performed ultrastructural analysis by transmission electron microscopy. For this purpose, human sperm from seronegative healthy donors were exposed in vitro for 3 and 6 h to infectious SARS-CoV-2 at 0.1 MOI. Transmission electron microscopy of sperm from seronegative donors that had been incubated with SARS-CoV-2 showed sperm-associated, virus-like particles similar to those previously described [1,24,25]; moreover, some infected cells showed cytopathological alterations throughout the entire cell. In particular, vesicles into the cytoplasm resembling the double-membrane vesicles (DMV), which are the subcellular site of viral replication, were often detected in infected cells (Figure 6). Finally, large intracellular virus-containing vesicles (LVCV), either connecting to the plasma membrane or not, were present.

**Figure 7:**
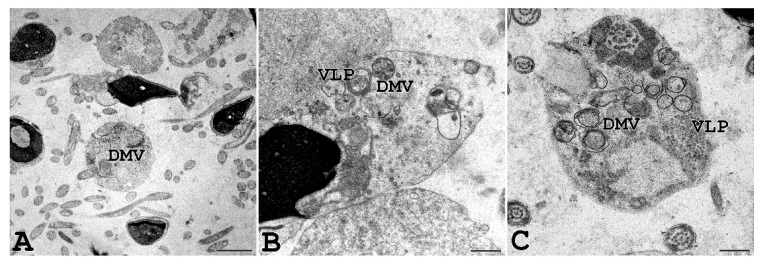
Ejaculated human sperm of a man affected by COVID-19. Human sperm from a donor man with a symptomatic form of COVID-19 were analysed, and virus-like particles (VLP) can be seen inside double-membrane vesicles (DMV) localized in the cytoplasmic residues embedding sperm tails, often coiled inside. Scale bar: (**A**) = 0.2 µm; (**B**,**C**) = 0.5 µm.

Interestingly, we were able to perform TEM analysis on the ejaculated semen of a COVID-19-affected man with a positive molecular swab and several symptoms, including fever, cough, nasal congestion, headache, and muscle pain. As shown in Figure 7, vesicles resembling DMV, known as the subcellular site of viral replication, were present in the sperm cytoplasmic residues, and virus-like particles were observed inside DMV.

## 4. Discussion

A growing body of evidence supports the idea that SARS-CoV-2 infection directly and indirectly impacts male reproductive function [26,27,28]; however, conflicting results advise further investigation.

This study definitively proves that SARS-CoV-2 is able to infect ejaculated human sperm both in vivo and in vitro and provides insights into the mechanisms of virus entry into the male gonad. Indeed, we demonstrated that ACE2, the host receptor for the viral spike protein, is expressed by Leydig but not by Sertoli cells, as suggested by several authors [9,29]. SARS-CoV-2 tropism for Leydig cells is in agreement with the decreased blood levels of testosterone in COVID-19 patients compared with healthy individuals and with the negative correlation between COVID-19 severity and total testosterone blood level [30,31,32]. Indeed, replication of SARS-CoV-2 into Leydig cells might disrupt testosterone synthesis and secretion at the testicular level, thus explaining the recurrence of hypogonadotropic hypogonadism in patients with COVID-19 [33]. The tropism of SARS-CoV-2 for Leydig cells is also confirmed by post mortem analysis demonstrating that testes from COVID-19 patients exhibited significant seminiferous tubular injury and reduced levels of Leydig cells [34]. This specific viral route of entry we demonstrated with our study lets us hypothesize a possible mechanism by which SARS-CoV-2 may affect spermatogenesis; indeed, Leydig cell damage is associated with disrupted spermatogenesis [35]. The ACE2 receptor and its short isoform were recently shown to be expressed in human spermatozoa [36]. In this study, we not only confirmed this data but also analysed the expression of the specific host proteases TMPRSS2, CSTL, and BSG, known to be involved in the cleavage of the S protein, which activates ACE2-mediated virus entry. We demonstrated a very low level of TMPRSS2 mRNA in sperm and a barely undetectable expression in the testis. This is partially in agreement with previous data demonstrating that TMPRSS2 is mainly expressed in spermatogonial stem cells and in spermatids [37]. However, it is now well established that SARS-CoV-2 entry into host cells is not only mediated by membrane fusion. Indeed, in the absence of TMPRSS2, the virus, once bound to ACE-2, can use a different entry pathway based on endocytosis and subsequent activation by cathepsin in clathrine vesicles [38]. Based on the expression of the alternative co-receptors BSG and CTSL, as shown in this work, we assume that these molecules play a key role in spike proteolysis, as already proposed by single-cell transcriptomic analysis [39]. Of relevance, the expression of these alternative host factors in the seminiferous epithelium, likely in the Sertoli cells, suggests an additional mechanism by which SARS-CoV-2 may detrimentally affect human spermatogenesis.

We demonstrated that the viral particles are able to adhere to sperm cells and detected the viral spike and nucleocapside antigens at the midpiece of the sperm tail, particularly in the presence of cytoplasmic residues in this region. Notably, by using transmission electron microscopy, we demonstrated that sperm cells are susceptible to SARS-CoV-2 infection and provided evidence for the multistep process of viral replication inside male gametes. Indeed, DMV, the large cytoplasmic vesicles where RNA replication occurs, were clearly visible. At the same time, our micrographs clearly show the cytoplasmic vesicles, with some containing a large number of virions (LVCV) and others containing only one virus particle (SVCV) [25]. Our data seem to disagree with a previously published paper reporting that among 32 COVID-19-affected men, only 1 presented positive SARS-CoV-2 PCR in semen and seminal plasma fractions; additionally, in this case, the viral culture was negative [18]. However, it should be noted that the ddPCR assay we used to assess the presence of viral genome could have been more sensitive compared to the standard RT-PCR; moreover, the authors of this study disclosed that samples were frozen and thawed twice before viral culture testing; therefore, this result should be interpreted with caution.

By investigating seminal parameters of 120 post-COVID-19 patients, a recent study demonstrated significant reductions in sperm concentration, the number of spermatozoa produced, and both total and progressive motility of the spermatozoa, despite the fact that viral RNA could not be detected in the semen of recovered men [15]. This is not surprising since only very low tiers of SARS-CoV-2 have been detected so far in non-respiratory sites [14]. Therefore, it cannot be excluded that the entry of SARS-CoV-2 into human testis and sperm during active infection induces significant damage lasting at least 3 months, the mean time of a spermatogenetic wave in humans.

## 5. Conclusions

The effect of the COVID-19 pandemic on male fertility is still debated. Here, by using a multidisciplinary approach, we demonstrated that SARS-CoV-2 can infect ejaculated sperm and provided evidence for the specific localization of molecules mediating SARS-CoV-2–host interactions in the male genital tract. These results might be of translational importance, contributing to the development of preventive and therapeutic strategies to avoid SARS-CoV-2 transmission by the sperm, both in vivo and in vitro, and manage reproduction-related consequences of COVID-19.

## Data Availability

All data underlying the study are available from the corresponding authors.

## Supplementary Materials

The following supporting information can be downloaded at: https://www.mdpi.com/article/10.3390/cells11172631/s1, Table S1: PrimePCR^TM^ Expression Probe Assay, specific Reaction for Droplets Digital Polymerase Chain; Table S2: List of antibodies used in this study.
