# Supplementary material for: Cellular and Molecular Mechanisms of In Vivo and In Vitro SARS-CoV-2 Infection: A Lesson from Human Sperm
Source: Cells. 2022 Aug 24;11(17):2631. doi: 10.3390/cells11172631 (PMC9455059; doi:10.3390/cells11172631)
Supplement: Supplementary file 1 [file cells-11-02631-s001.zip › Supplementary material Table S1.pdf]

Supplementary Materials

Table S1. ID and sequences of primer used to detect gene expression

**Target Genes**	**Acronym**	**ID assay/primer sequence**
Angiotensin-converting enzyme 2	ACE2	Hs.PT.58.27645939
Transmembrane serine protease 2	TMPRSS2	Hs.PT.58.39408998
Cathepsin L	CTSL	Hs.PT.58.45751460
Basigin	BSG	Hs.PT.56a.39293590.g
Spike	SPIKE	5′GGCACGTAGTGTAGCTAGTC3′<br>5′TGGGTATGGCAATAGAGTTATTAG3<br>5′FAM-ACTATGTCACTTGGT-MGB3′
**Reference Genes**	**Acronym**	**ID assay**
HypoxanthineGuanine PhosphoribosylTransferase 1	HPRT1	Hs.PT.58v.45621572
Glyceraldehyde 3-phosphate dehydrogenase	GAPDH	Hs.PT.58.589810.g
