# Supplementary material for: Cellular and Molecular Mechanisms of In Vivo and In Vitro SARS-CoV-2 Infection: A Lesson from Human Sperm
Source: Cells. 2022 Aug 24;11(17):2631. doi: 10.3390/cells11172631 (PMC9455059; doi:10.3390/cells11172631)
Supplement: Supplementary file 1 [file cells-11-02631-s001.zip › Supplementary material Table S2.pdf]

Supplementary Materials

Table S2. List of antibodies used in this study

Antigen	Donor species	Dilution	Manufacturer	RRID
Primary antibodies				
ACE-2	Mouse	1:250	R&D System	AB_355722
TMPRSS2	Mouse	1:200	Santa Cruz Technology	AB_2205599
CTSL	Mouse	1:500	Santa Cruz Technology	AB_626811
BSG	Mouse	1:500	Santa Cruz Technology	AB_626911
SARS-CoV2 Nucleocapsid	Mouse	1:1000	Bio-techne	AB_1522790
SARS-CoV2 Spike	Rabbit	1:1000	Bio-techne	AB_1237374
INSL3	Rabbit	1:100	Thermo Fisher Scientific	AB_2720534
Secondary antibodies				
Anti-Mouse IgM FITC	Goat	1:500	Sigma Life Science	AB_259799
Anti-Rabbit IgM FITC	Goat	1:200	Sigma Life Science	AB_1137637
Anti-Rabbit IgG TRITC	Goat	1:1000	Thermo Fisher Scientific	AB_2534775
